# The beneficial effect of Escalated‐R‐CHOP‐21 for the treatment of diffuse large B‐cell lymphoma in elderly male patients: A propensity‐matched cohort study

**DOI:** 10.1002/cam4.4296

**Published:** 2021-09-28

**Authors:** Hai‐Xia He, Yan Gao, Bing Bai, Xiao‐Xiao Wang, Ji‐Bin Li, Cheng Huang, Jia‐Ying Mao, Li‐Qin Ping, Qi‐Xiang Rong, Yan‐Xia He, He Huang, Qing‐Qing Cai, Zhi‐Ming Li, Wen‐Qi Jiang, Hui‐Qiang Huang

**Affiliations:** ^1^ State Key Laboratory of Oncology in South China & Collaborative Innovation Center of Cancer Medicine Sun Yat‐sen University Cancer Center Guangzhou China; ^2^ Department of Medical Oncology Sun Yat‐sen University Cancer Center Guangzhou China; ^3^ Department of Clinical Research Sun Yat‐sen University Cancer Center Guangzhou China

**Keywords:** diffuse large B‐cell lymphoma, elderly males, escalated, R‐CHOP, rituximab, standard

## Abstract

**Purpose:**

Some studies have indicated that using 500 mg/m^2^ rituximab combined with CHOP‐14 may be beneficial for elderly men but not women with diffuse large B‐cell lymphoma (DLBCL). The purpose of this study was to investigate the potential benefit of escalated doses of rituximab with CHOP‐21 as the first‐line treatment in male patients with DLBCL.

**Methods:**

We performed a retrospective cohort study to analyze the survival benefit of rituximab 500 mg/m^2^ plus the CHOP‐21 regimen (Escalated‐R‐CHOP‐21) as the first‐line treatment compared with using rituximab 375 mg/m^2^ plus the CHOP‐21 regimen (Standard‐R‐CHOP‐21) in men with DLBCL. We used propensity score matching to maximize the balance of the observed covariables. The primary endpoints of this study were the progression‐free survival (PFS) rate and overall survival (OS) rate at 3 years.

**Results:**

After a median follow‐up of 47 months (IQR 31–65), no significant difference in PFS and OS was found for men treated with Escalated‐R‐CHOP‐21 compared with Standard‐R‐CHOP‐21 [3‐year PFS: 69.7% versus 71.9%, *p* = 0.867; 3‐year OS: 83.0% versus 82.4%, *p* = 0.660]. After 1:1 propensity score matching, we found that the patients using Escalated‐R‐CHOP‐21 had statistically significant survival benefits relative to Standard‐R‐CHOP‐21 among the 96 matched elderly male patients for 3‐year PFS [75.5% (95% CI 62.8–88.2) versus 58.2% (95% CI 44.3–72.1); *p* = 0.019] and 3‐year OS [86.6% (95% CI 76.4–96.8) versus 65.8% (95% CI 52.1–79.5); *p* = 0.017]. However, no differences in survival were observed for younger male patients. Furthermore, the dose effect in PFS of Escalated‐R‐CHOP‐21 was more obvious for elderly male patients with no high‐risk extranodal sites (*p* = 0.005 and interaction *p* = 0.030).

**Conclusion:**

Escalated‐R‐CHOP‐21 could be a safe and effective option for treating elderly male patients with DLBCL. This study provides new insight into optimizing the standard treatment regimen, which may have important therapeutic implications in elderly male patients with DLBCL.

## Graphical abstract

In this study, we investigated the potential benefit of rituximab 500 mg/m^2^ plus the CHOP‐21 regimen (Escalated‐R‐CHOP‐21) as the first‐line treatment compared with using rituximab 375 mg/m^2^ plus the CHOP‐21 regimen (Standard‐R‐CHOP‐21) in male patients with DLBCL. We found that only elderly men had statistically significant survival benefits from Escalated‐R‐CHOP‐21 than Standard‐R‐CHOP‐21 of 3‐year PFS [75.5% (95% CI 62.8–88.2) vs. 58.2% (95%CI 44.3–72.1); *p* = 0.019] and 3‐year OS [86.6% (95% CI 76.4–96.8) vs. 65.8.0% (95% CI 52.1–79.5); *p* = 0.017] without causing severe toxicity after propensity score matching.

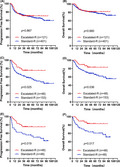

## 1 INTRODUCTION

Adding rituximab (R) to the conventional CHOP (R‐CHOP) regimen for diffuse large B‐cell lymphoma (DLBCL) has significantly improved its efficacy and the survival rate by 10%–20%.^1^, ^2^, ^3^ Although R‐CHOP has gradually become the standard treatment of DLBCL, approximately 20%–30% of patients still relapse or are refractory.^4^, ^5^ Identifying patients with a poor prognosis and optimizing treatment strategies are essential to improve the outcome of DLBCL.

Elderly men with DLBCL who are treated with R‐CHOP are more likely to have poor outcomes than those treated with CHOP alone.^6^ The common dose (375 mg/m^2^) of rituximab used for DLBCL is suboptimal for elderly men due to the faster rituximab plasma clearance.^7^ The optimal dose of rituximab still needs to be defined because the 375 mg/m^2^ dose of rituximab is mostly based on industrial consideration.^8^ The SEXIE‐R‐CHOP‐14 trial indicated that 2 weeks of rituximab 500 mg/m^2^ with CHOP‐14 may alleviate the unfavorable prognosis of elderly men.^9^ Prolonging the exposure time to rituximab was associated with better PFS and OS of elderly men in the SMARTE‐R‐CHOP‐14 trial.^10^ Moreover, these studies also indicated that using 500 mg/m^2^ rituximab combined with CHOP‐14 may be more beneficial in elderly men than in elderly women.^9^, ^10^ Based on these data, in NCCN guidelines, a rituximab dose of 500 mg/m^2^ was recommended for men >60 years of age treated with R‐CHOP.

In addition, the MInT study found that younger men benefited from the addition of rituximab to the same extent as younger women.^11^ The median rituximab clearance in younger men (9.89 ml/h) was significantly higher than that in elderly women (8.47 ml/h, *p* = 0.015).^12^ Whether younger men can obtain a better outcome with increased rituximab doses remains unknown, and no studies have been conducted specifically of increased rituximab doses in younger men.

On the other hand, R‐CHOP‐21 is non‐inferior to R‐CHOP‐14 in terms of efficacy as a first‐line treatment of DLBCL.^13^ The main advantage of R‐CHOP‐21, administered for 21 days of each cycle, is its efficient, convenient applications, and its tolerable safety profile. It is meaningful to explore the efficacy and safety of high‐dose rituximab combined with CHOP‐21 in male patients with DLBCL. To evaluate this clinical issue, we used a propensity‐matched method to assess whether male patients would benefit from high‐dose rituximab combined with CHOP‐21.

## 2 MATERIALS AND METHODS

### 2.1 Patient inclusion

Upon approval by the Institutional Ethical Board of Sun Yat‐sen University Cancer Center (ethics approval number: B2020‐279‐01), we performed a retrospective cohort study to analyze the difference in the survival benefit between male patients with DLBCL who received at least two cycles of rituximab 500 mg/m^2^ combined with CHOP‐21 (Escalated‐R‐CHOP‐21) or the rituximab 375 mg/m^2^ combined with CHOP‐21 (Standard‐R‐CHOP‐21) as their first‐line chemoimmunotherapy regimen. All patients were fully informed of the available treatment option, and part of the patients chosen the escalated rituximab dosage combined with CHOP‐21 after understanding the potential benefit. The corresponding treatment consent was signed and stored in the medical record. The informed consent for patients enrolled in this study was waived by the ethics committee because of its retrospective nature. In this study, 201 elderly men (age >60 years) and 321 younger men (18–60 years) were included. After 1:1 propensity score matching, there were 96 elderly men and 146 younger men in the matched cohort, respectively (see Figure S1). The inclusion criteria included age 18 years or older and newly diagnosed, CD20‐positive, biopsy‐confirmed DLBCL. Female patients were excluded from this study, and male patients were also excluded if they had previously been treated, had CNS lymphoma, HIV infection, or had a history of other lymphomas or malignancies. Furthermore, the patients should have normal cardiopulmonary, pulmonary, hepatic and renal function, performance status under 2, initial WBC over 2.5 × 10^9^/L, and initial platelet over 100 × 10^9^/L unless bone marrow involvement was present. Patients who had incomplete data or were lost to follow‐up were ineligible.

### 2.2 Evaluation and treatment

Patients underwent mandatory baseline assessments including clinical examination, relevant laboratory tests, positron emission tomography scans (PET scans) or computed tomography scans (CT scans) of the whole body, and a bone marrow biopsy. Patients’ information was collected from medical records. All patients were staged using the Ann Arbor staging system and stratified according to the International Prognostic Index (IPI),^14^ and an enhanced IPI derived from the National Comprehensive Cancer Network (NCCN) database (NCCN‐IPI),^15^ and a prognostic model to predict the risk of central nervous system (CNS) relapse incorporating five indexes of IPI plus kidney or adrenal gland involvement (CNS‐IPI).^16^ We categorized the primary sites of the DLBCL as nodal, extranodal high‐risk, or other extranodal. High‐risk extranodal sites were defined by prior studies in DLBCL in the rituximab era, which included the central nervous system, lung, liver, pancreas, gastrointestinal tract, and bone marrow.^17^ CNS prophylaxis was usually recommended in patients with a high‐risk of CNS‐IPI, which included intrathecal (IT) or high‐dose methotrexate (HD‐MTX).^18^

The treatment of Standard‐R‐CHOP‐21 consisted of rituximab 375 mg/m^2^, cyclophosphamide (CTX) 750 mg/m^2^, doxorubicin 50 mg/m^2^, or equivalent doses of other anthracyclines (ANT), vincristine 1.4 mg/m^2^ [capped at 2.0 mg], all given intravenously on day 1 and oral prednisone 100 mg on days 1–5 delivered in a 21‐day cycle.^13^ The Escalated‐R‐CHOP‐21 regimens included these same therapeutic agents except for the dose of rituximab administered was 500 mg/m^2^. A split‐dose regimen or dose reductions of R‐CHOP were allowed to ameliorate the adverse effects during the chemotherapy cycle, and symptomatic treatment was carried out according to the condition of the patients. Thirty cases (5.7%) of dose reduction happened in 522 patients and 9.0% (18/201) in the elderly men. Among the elderly men, there are 14.6% (7/48) in the Escalated‐R group and 7.2% (11/153) in the Standard‐R group. Tumor lysis prophylaxis, antiemetics, granulocyte colony‐stimulating factor, and supportive care were administered at the discretion of the physician. Radiotherapy applied to initial bulky disease or extranodal involvement was allowed. All patients underwent imaging evaluation every two cycles until the end of chemotherapy.

### 2.3 Follow‐up and outcome

The primary endpoints of this study were progression‐free survival (PFS) and overall survival (OS). PFS was defined from the date of first treatment until the date of disease progression, relapse, death from any cause, or the time of the last follow‐up. OS was calculated as the period from the first treatment until death or the last follow‐up. The key secondary endpoints were the objective response rate (ORR) of the disease, which includes complete response (CR) and partial response (PR) evaluated by the physician and by central imaging review. The efficacy assessment referred to the 2014 Cheson Lugano criteria.^19^ We also explored the relationship between CNS relapse and treatment regimen. CNS relapse was calculated as the period from the first treatment until relapse in the CNS.

### 2.4 Statistical analysis

To control for baseline imbalances, we used propensity score matching to maximize the balance of the observed covariables.^20^ The propensity to undergo Escalated‐R versus Standard‐R was estimated using a logistic regression model based on stage, LDH, extranodal sites, and IPI. The matching algorithm was 1:1 matched with no replacement. The matching caliper in the elderly and younger male cohorts was 0.001. The patients’ characteristics and outcomes were compared between the two groups before and after matching. PFS and OS were estimated using the Kaplan–Meier method and were compared using the log‐rank test. Univariate analysis was performed using a Cox proportional hazards regression model to determine the prognostic value. A multivariate analysis focusing on rituximab dosage was performed using a Cox regression model. Subgroup analyses were performed to assess the homogeneity of the association between the treatment survival among different subgroups of patients. Effects and interaction *p* values were calculated. All statistical analyses were two‐sided and were performed using SPSS version 24.0 (IBM Corporation).

## 3 RESULTS

### 3.1 Clinical characteristics

Between 11 January 2011 and 17 May 2019, 522 male patients with newly diagnosed DLBCL were included in this study. The number of elderly and younger men was 201 (38.5%) and 321 (61.5%), respectively, and 121 male patients were treated with Escalated‐R‐CHOP‐21. The last day of follow‐up (and the data cutoff date) was 1 September 2020. The median follow‐up period was 47 months (IQR 31–65). The baseline clinical characteristics of all male patients and the elderly male cohort are summarized in Table 1, and the characteristics of the younger male cohort are listed in Table S1. The patients treated with Escalated‐R‐CHOP‐21 (Escalated‐R group) and treated with Standard‐R‐CHOP‐21 (Standard‐R group) were similar in median age in the unmatched elderly male cohort (68.2 vs. 69.1 years, *p* = 0.373), and the same results were obtained in the unmatched younger male cohort treated with the above regimen (44.8 vs. 43.4 years, *p* = 0.326). The median number of treatment cycles was 6 (range 2–8) in the different groups. The distribution of normal LDH was significantly different between the Escalated‐R group (52.1%) and the Standard‐R group (65.7%) in the younger male cohort (*p* = 0.034). In the unmatched elderly male cohort, the Escalated‐R group had a lower proportion of high‐risk IPI (14.6% vs. 22.2%, *p* = 0.227) and had a lower high‐risk extranodal sites (20.8% vs. 37.3%, *p* = 0.099) than the Standard‐R group. Aside from these exceptions, there was no discrepancy between the Escalated‐R group and Standard‐R group regarding baseline data in each cohort. After 1:1 propensity score matching, a good balance in some variations (stage, LDH, extranodal sites, PS, and IPI) was achieved for the two matched cohorts (*p* = 1).

**TABLE 1:** Baseline numbers and participant characteristics of whole male patients and the elderly male cohort

Parameters	Whole population			Unmatched elderly cohort			Matched elderly cohort		
	Standard‐R (*n* = 401)	Escalated‐R (*n* = 121)	*p* value	Standard‐R (*n* = 153)	Escalated‐R (*n* = 48)	*p* value	Standard‐R (*n* = 48)	Escalated‐R (*n* = 48)	*p* value
Age (y)	53.2 ± 15.9	54.1 ± 14.9	0.568	69.1 ± 6.0	68.2 ± 6.5	0.373	68.5 ± 5.5	68.2 ± 6.5	0.800
Stage									
I–II	222 (55.4)	64 (52.9)	0.632	74 (48.4)	24 (50.0)	0.843	24 (50.0)	24 (50.0)	1
III–IV	179 (44.6)	57 (47.1)		79 (51.6)	24 (50.0)		24 (50.0)	24 (50.0)	
Extranodal sites									
≤1	297 (74.1)	91 (75.2)	0.801	104 (68.0)	38 (79.2)	0.137	38 (79.2)	38 (79.2)	1
>1	104 (25.9)	30 (24.8)		49 (32.0)	10 (20.8)		10 (20.8)	10 (20.8)	
PS									
0–1	372 (92.8)	113 (93.4)	0.816	138 (90.2)	45 (93.8)	0.644	45 (93.8)	45 (93.8)	1
2–4	29 (7.2)	8 (6.6)		15 (9.8)	3 (6.3)		3 (6.3)	3 (6.3)	
LDH (U/L)									
≤250	259 (64.6)	67 (55.4)	0.067	96 (62.7)	29 (60.4)	0.772	29 (60.4)	29 (60.4)	1
>250	142 (35.4)	54 (44.6)		57 (37.3)	19 (39.6)		19 (39.6)	19 (39.6)	
B symptom									
No	332 (82.8)	96 (79.3)	0.386	128 (83.7)	40 (83.3)	0.957	38 (79.2)	40 (83.3)	0.601
Yes	69 (17.2)	25 (20.7)		25 (16.3)	8 (16.7)		10 (20.8)	8 (16.7)	
Bulky disease (cm)									
≤5	284 (70.8)	89 (73.6)	0.719	115 (75.2)	37 (77.1)	0.564	31 (64.6)	37 (77.1)	0.394
5 < *x* ≤ 10	83 (20.7)	21 (17.4)		29 (19.0)	10 (20.8)		15 (31.3)	10 (20.8)	
>10	34 (8.5)	11 (9.1)		9 (5.9)	1 (2.1)		2 (4.2)	1 (2.1)	
Cell of origin									
GCB	168 (41.9)	50 (41.3)	0.504	67 (43.8)	16 (33.3)	0.363	18 (37.5)	16 (33.3)	0.740
Non‐GCB	218 (54.4)	69 (57.0)		81 (52.9)	31 (64.6)		28 (58.3)	31 (64.6)	
Unclassified	15 (3.7)	2 (1.7)		5 (3.3)	1 (2.1)		2 (4.2)	1 (2.1)	
Primary sites									
Nodal	193 (48.1)	60 (49.6)	0.655	57 (37.3)	24 (50.0)	0.099	21 (43.8)	24 (50.0)	0.508
Extranodal, high‐risk	129 (32.2)	34 (28.1)		57 (37.3)	10 (20.8)		15 (31.2)	10 (20.8)	
Extranodal, other	79 (19.7)	27 (22.3)		39 (25.5)	14 (29.2)		12 (25.0)	14 (29.2)	
CNS prophylaxis									
No	358 (89.3)	105 (86.8)	0.447	137 (89.5)	39 (81.3)	0.129	41 (85.4)	39 (81.3)	0.584
Yes	43 (10.7)	16 (13.2)		16 (10.5)	9 (18.7)		7 (14.6)	9 (18.7)	
CNS‐IPI									
Low	230 (57.4)	68 (56.2)	0.227	57 (37.3)	21 (43.8)	0.363	21 (43.8)	21 (43.8)	0.837
Intermediate	111 (27.7)	41 (33.9)		59 (38.6)	20 (41.7)		18 (37.5)	20 (41.7)	
High	60 (15.0)	12 (9.9)		37 (24.2)	7 (14.6)		9 (14.3)	7 (14.6)	
IPI									
Low	230 (57.4)	68 (56.2)	0.164	57 (37.3)	21 (43.8)	0.227	21 (43.8)	21 (43.8)	1
LI	71 (17.7)	16 (13.2)		32 (20.9)	6 (12.5)		6 (12.5)	6 (12.5)	
HI	58 (14.5)	27 (22.3)		30 (19.6)	14 (29.2)		14 (29.2)	14 (29.2)	
High	42 (10.5)	10 (8.3)		34 (22.2)	7 (14.6)		7 (14.6)	7 (14.6)	
NCCN‐IPI									
Low	112 (27.8)	32 (26.4)	0.950	0	0	0.529	0	0	0.898
LI	188 (47.1)	57 (47.1)		75 (49.0)	28 (58.3)		26 (54.2)	28 (58.3)	
HI	85 (21.1)	28 (23.1)		63 (41.2)	16 (33.3)		17 (35.4)	16 (33.3)	
High	16 (4.0)	4 (3.3)		15 (9.8)	4 (8.3)		5 (10.4)	4 (8.3)	

Note

Data of age are means ± standard deviations and *p* value computed using independent sample *t*‐test for continuous variables. Numbers of patients with percentages in parentheses and *p* value computed using the chi‐squared test or Fisher’s exact test for categorical variables. LDH, lactic dehydrogenase; PS, performance state. CNS‐IPI: Low‐risk: 0–1, Intermediate‐risk: 2–3, High‐risk: 4–6 or kidney or adrenal gland involvement. IPI: Low‐risk: 0–1, Intermediate low (LI): 2, Intermediate high (HI): 3, High‐risk: ≥4. NCCNIPI: Low‐risk: 0–1, Intermediate low (LI): 2–3, Intermediate high (HI): 4–5, High‐risk: ≥6.

### 3.2 Response to therapy

Tumor response was assessed by the physician at the end of therapy, and the data are shown in Table 2. In the elderly male cohort, compared with the matched Standard‐R group, the Escalated‐R group had a significantly higher CR rate (81.3% vs. 62.5%; odds ratio, 2.60; 95% CI, 1.03–6.60; *p* = 0.041). However, there were no significant differences in ORR between the Escalated‐R group and the Standard‐R group in the matched elderly male cohort (93.8% vs. 91.7%). Although the ORR of the Escalated‐R group was lower than the Standard‐R group in the matched younger male cohort (89.0% vs. 97.3%), the difference was not statistically significant (odds ratio, 0.23; 95% CI, 0.05–1.12; *p* = 0.097). Taken together, these results indicated that elderly men benefited from the Escalated‐R regimen, especially in terms of the CR rate.

**TABLE 2:** Response to therapy in the elderly and the younger male cohorts

	Elderly male cohort			Younger male cohort		
	Unmatched standard‐R (*n* = 153)	Matched standard‐R (*n* = 48)	Escalated‐R (*n* = 48)	Unmatched standard‐R (*n* = 248)	Matched standard‐R (*n* = 73)	Escalated‐R (*n* = 73)
ORR	139 (90.9%)	44 (91.7%)	45 (93.8%)	238 (96.0%)	71 (97.3%)	65 (89.0%)
CR	108 (70.6%)	30 (62.5%)	39 (81.3%)	189 (76.2%)	53 (72.6%)	53 (72.6%)
PR	31 (20.3%)	14 (29.2%)	6 (12.5%)	49 (19.8%)	18 (24.7%)	12 (16.4%)
SD	3 (2.0%)	2 (4.2%)	0	0	0	0
PD	7 (4.6%)	2 (4.2%)	3 (6.3%)	7 (2.8%)	2 (2.7%)	6 (8.2%)
NA	4 (2.6%)	0	0	3 (1.2%)	0	2 (2.7%)

Abbreviations: CR, complete response; ORR, objective complete response; PD, progressive disease; PR, partial response; NA, not assessable; SD, stable disease.

### 3.3 Survival outcomes

**FIGURE 1:**
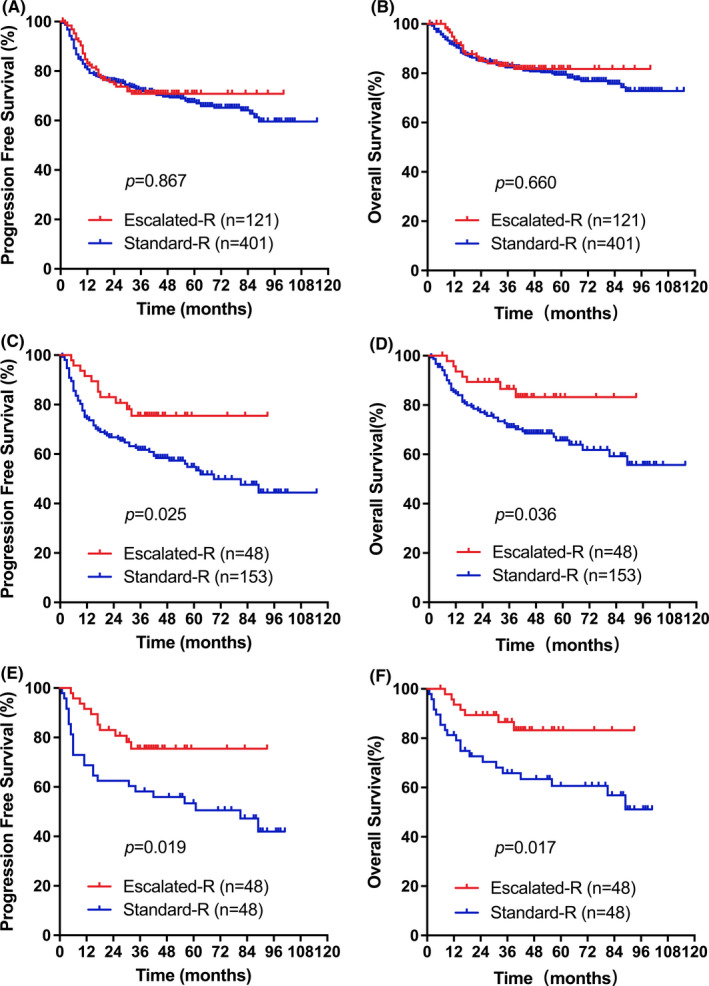
The outcome for the Escalated‐R group and Standard‐R group in whole male patients and the elderly male cohort. For A to B, PFS and OS in whole male patients (*n* = 522). For C to D, PFS and OS in the elderly male cohort (*n* = 201). For E to F, PFS and OS in the matched elderly male cohort (*n* = 96)

After a median follow‐up of 40 months (IQR 24–48) in the Escalated‐R group and 50 months (IQR 34–72) in the Standard‐R group, no significant difference in PFS was observed among all male patients treated with Escalated‐R‐CHOP‐21 compared with patients treated with Standard‐R‐CHOP‐21 [3‐year PFS: 69.7% vs. 71.9%; *p* = 0.867] (Figure 1A). Similarly, there was no significant difference in OS [3‐year OS: 83.0% vs. 82.4%; *p* = 0.660] (Figure 1B). The results were different when the patients were divided into the elderly male cohort and the younger male cohort. In the elderly male cohort, there were significant differences in 3‐year PFS (75.5% (95% CI 62.8–88.2) vs. 61.7% (95% CI 53.9–69.5); *p* = 0.025) and 3‐year OS (86.6% (95% CI 76.4–96.8) vs. 71.1% (95% CI 63.7–78.5); *p* = 0.036) between the Escalated‐R group and the unmatched Standard‐R group (Figure 1C,D). The same trend was observed in the matched datasets consisting of 96 elderly men after performing propensity score matching (Figure 1E,F). We found that the Escalated‐R group had statistically significant survival benefits compared with the matched Standard‐R group in 3‐year PFS [75.5% (95% CI 62.8–88.2) vs. 58.2% (95% CI 44.3–72.1); *p* = 0.019] and 3‐year OS [86.6% (95% CI 76.4–96.8) vs. 65.8% (95% CI 52.1–79.5); *p* = 0.017] (Figure 1E,F). These results indicated that increasing the rituximab dose to 500 mg/m^2^ was associated with a survival benefit in elderly men.

**FIGURE 2:**
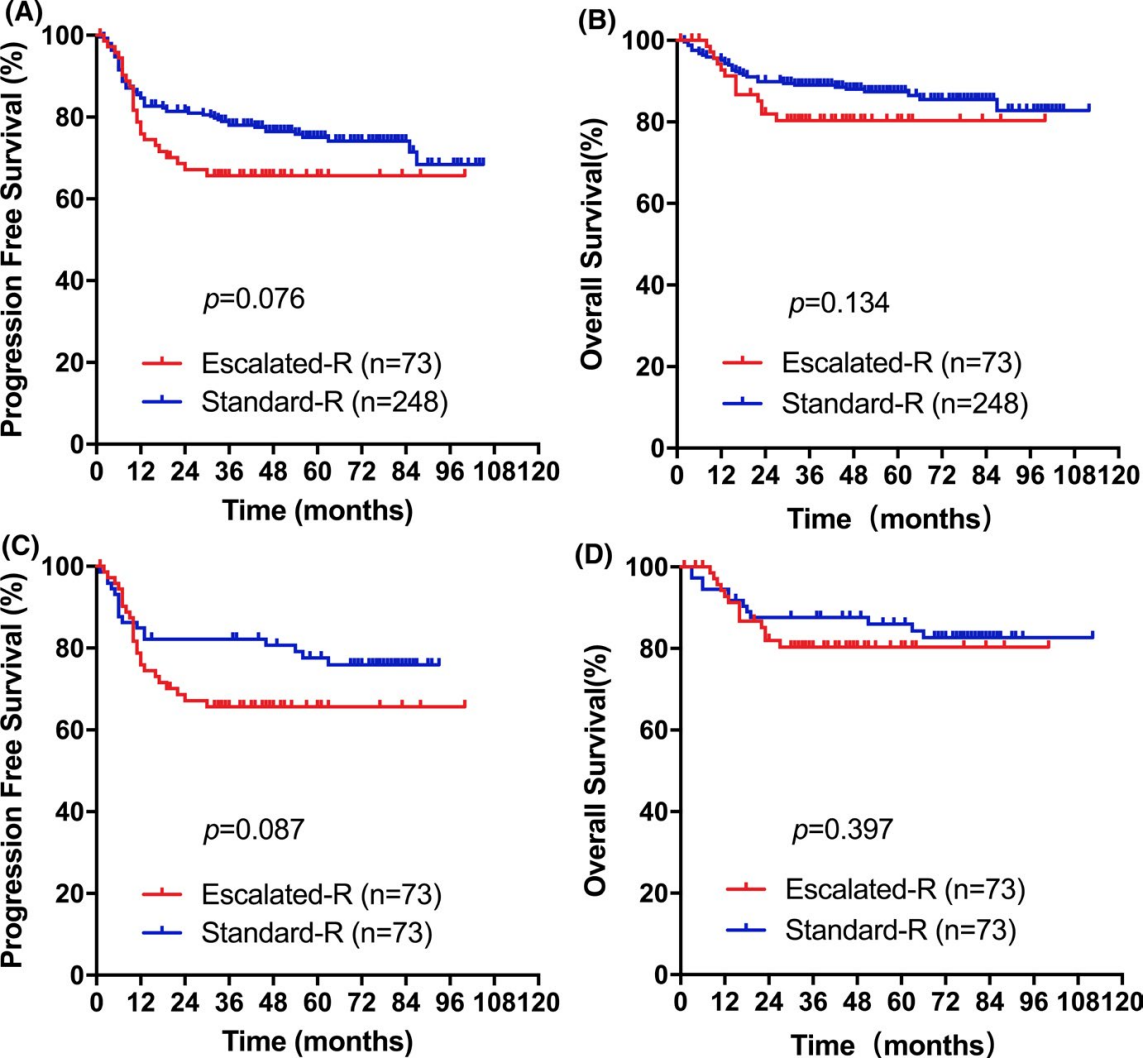
The outcome for the Escalated‐R group and Standard‐R group in the younger male cohort. For A to B, PFS and OS in the younger male cohort (*n* = 321). For C to D, PFS and OS in the matched younger male cohort (*n* = 146)

Conversely, no differences were observed between Escalated‐R‐CHOP‐21 and Standard‐R‐CHOP‐21 in the younger male cohort (Figure 2). In the unmatched younger male cohort, the 3‐year PFS was 65.6% (95% CI 54.4–76.8) for those in the Escalated‐R‐CHOP‐21 versus 78.0% (95% CI 72.9–83.1) for those in the Standard‐R group (*p* = 0.076), and the 3‐year OS was 80.4% (95% CI 70.8–90.0) versus 89.0% (95% CI 85.1–92.9) (*p* = 0.134), respectively (Figure 2A,B). After performing propensity score matching, the matched Standard‐R group had a 3‐year PFS rate of 82.2% (95% CI 73.4–91.0) and 3‐year OS rate of 87.6% (95% CI 80.0–95.2). There were no significant differences in a 3‐year PFS (*p* = 0.087) or 3‐year OS (*p* = 0.397) between the Escalated‐R group and the Standard‐R group in the matched datasets for the 146 younger men (Figure 2C,D). These results indicated that patients who received Standard‐R‐CHOP‐21 might have a trend of better PFS than Escalated‐R‐CHOP‐21 in the younger male cohort.

### 3.4 Risk factors associated with the outcomes of the patients

To explore the independent prognostic significance of the rituximab dose for elderly men, univariate and multivariate Cox regression analyses for PFS and OS were performed. Elderly men using Escalated‐R‐CHOP‐21 had significantly decreased risks of progression and death compared with those using Standard‐R‐CHOP‐21 in both univariate and multivariate analyses (Table 3), but insignificantly for younger men (Table S2). Univariate analysis in the elderly male cohort suggested that variables including the stage, extranodal sites, LDH level, CNS‐IPI, IPI, and NCCN‐IPI were prognostic factors of PFS and OS in addition to rituximab dosage, and bulky disease had a predictive value for PFS but not for OS (Table 3). We also investigated whether the disease site and CNS prophylaxis were associated with the prognosis of DLBCL patients. A trend was observed that high‐risk extranodal sites and CNS prophylaxis were associated with worse prognosis.

**TABLE 3:** Univariable and multivariable analyses of prognostic factors for PFS and OS in the elderly male cohort

	PFS		OS	
	HR (95%CI)	*p* value	HR (95%CI)	*p* value
*Univariate analysis*				
Stage	2.65	**<0.001**	2.42	**0.002**
III–IV vs. I–II	1.65–4.26		1.38–4.23	
Extranodal sites	1.97	**0.003**	1.84	**0.025**
>1 vs. ≤1	1.26–3.09		1.08–3.12	
PS	1.33	0.427	1.42	0.389
2–4 vs. 0–1	0.66–2.66		0.64–3.13	
LDH (U/L)	1.56	**0.047**	1.84	**0.021**
>250 vs. ≤250	1.01–2.42		1.10–3.08	
Bulky disease (cm)	1.51	**0.025**	1.43	0.081
>10 vs. 5–10 vs. ≤5	1.05–2.15		0.96–2.12	
B symptom	1.34	0.290	1.06	0.856
Yes vs. No	0.78–2.29		0.55–2.06	
Cell of origin	1.25	0.335	1.46	0.177
Non‐GCB vs. GCB	0.79–1.98		0.84–2.53	
Primary sites	1.39	0.156	1.06	0.826
Extranodal, high‐risk vs. others	0.88–2.17		0.62–1.83	
CNS prophylaxis	1.17	0.628	0.84	0.681
Yes vs. No	0.62–2.21		0.36–1.95	
CNS‐IPI	2.30	**0.002**	2.06	**0.019**
High vs. Low	1.37–3.85		1.13–3.76	
IPI	2.25	**<0.001**	2.21	**0.003**
High vs. Low	1.44–3.51		1.31–3.74	
NCCN‐IPI	2.91	**<0.001**	2.80	**<0.001**
High vs. Low	1.81–4.66		1.60–4.90	
R dosage	0.49	**0.030**	0.44	**0.043**
Escalated vs. Standard	0.26–0.93		0.20–0.97	
*Multivariate analysis with exposure* ^a^				
R dosage	0.50	**0.035**	0.43	**0.041**
Escalated vs. Standard	0.26–0.95		0.19–0.97	
*Multivariate analysis with IPI*				
R dosage	0.48	**0.024**	0.43	**0.035**
Escalated vs. Standard	0.25–0.91		0.19–0.94	
*Multivariate analysis with NCCN‐IPI*				
R dosage	0.52	**0.044**	0.47	0.060
Escalated vs. Standard	0.27–0.98		0.21–1.03	
*Multivariate analysis with CNS‐IPI*				
R dosage	0.52	**0.043**	0.46	0.055
Escalated vs. Standard	0.27–0.98		0.21–1.02	

Note

Abbreviations: PS, performance state; LDH, lactic dehydrogenase.

High and low CNS‐IPI was defined as the risk score 0‐1 and more than 2, respectively. High and low IPI was defined as the risk score 0‐2 and 3‐5, respectively. High and low NCCNIPI was defined as the risk score 0‐3 and more than 4, respectively. *p*‐value <0.05 in bold shows statistically significant.

a Represents variables with *p* value <0.1 in the univariate analysis except for CNS‐IPI, IPI and NCCNIPI.

The indexes of the CNS‐IPI, IPI, and NCCN‐IPI overlapped with other factors, such as stage, extranodal sites, PS, and LDH. In order to evaluate whether rituximab dosage was an independent prognostic factor, a multivariate analysis was performed with variables with *p* < 0.1 in the univariate analysis except for CNS‐IPI, IPI, and NCCNIPI. Then we also did other multivariate analysis that included rituximab dosage with CNS‐IPI, IPI, and NCCN‐IPI. The results showed that rituximab dosage was a significant independent predictor of both PFS and OS across all Cox regression models, but it was not statistically significant for predicting OS when combined with the CNS‐IPI or NCCN‐IPI in elderly men. However, the rituximab dosage was not associated with improved PFS or OS in younger men, and the results of the univariate and multivariate analyses are listed in Supplementary Table 2.

### 3.5 Subgroup analyses by important covariables

**FIGURE 3:**
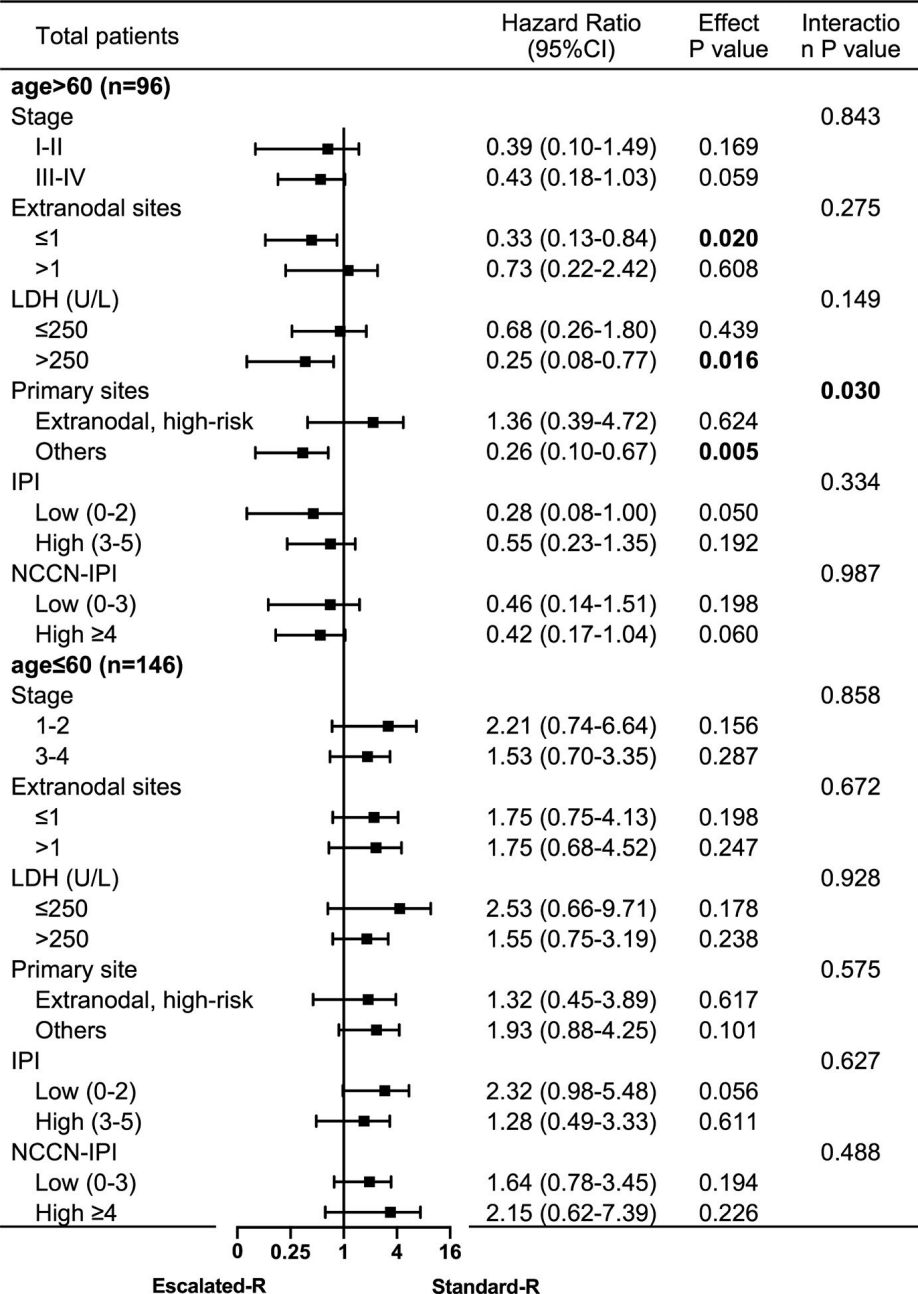
Forest plot for subgroup analyses of PFS according to the age of male patients. The Escalated‐R group was compared with the Standard‐R group in calculating hazard ratios and 95% confidence intervals

**FIGURE 4:**
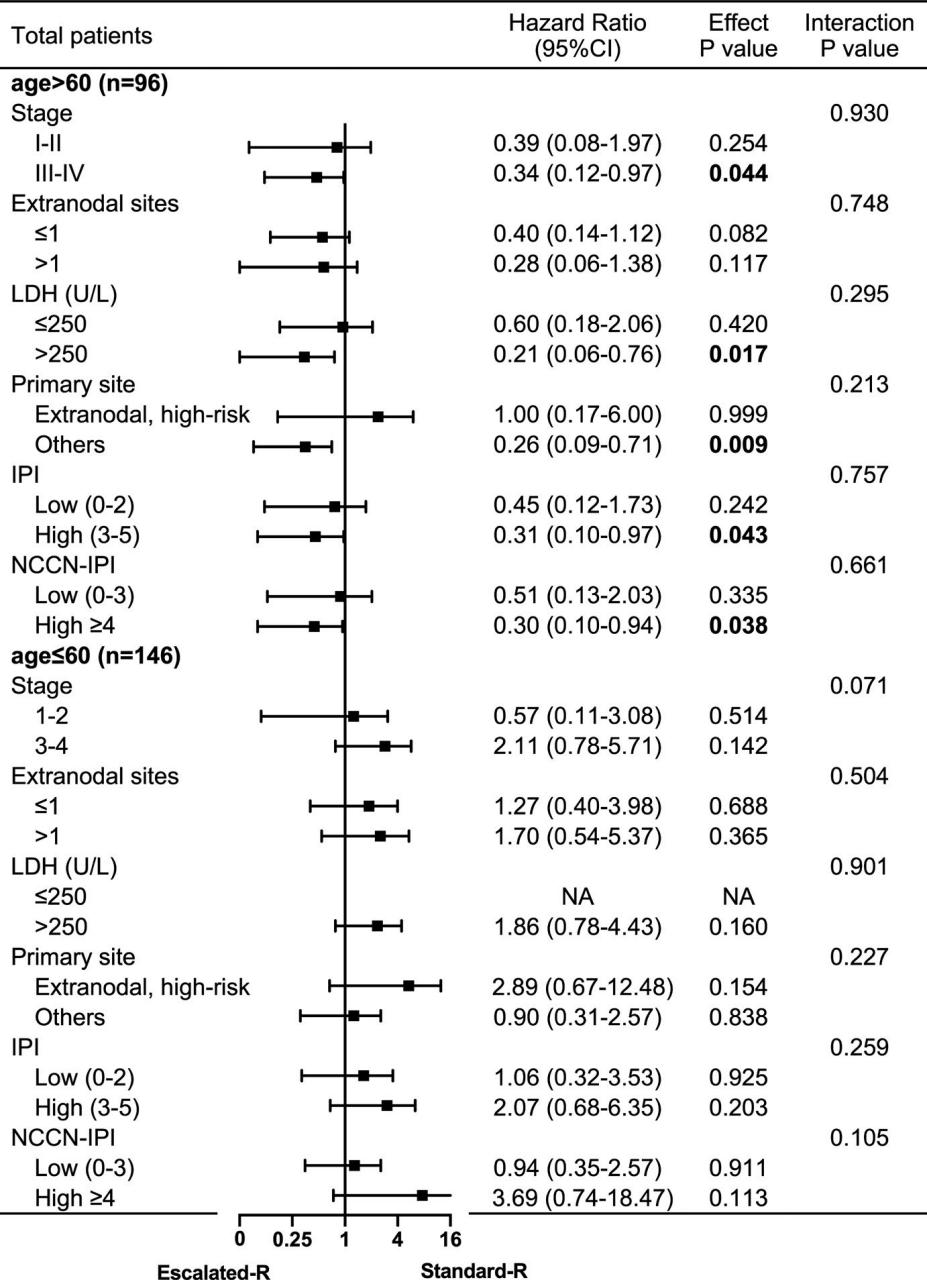
Forest plot for subgroup analyses of OS according to the age of male patients. The Escalated‐R group was compared with the Standard‐R group in calculating hazard ratios and 95% confidence intervals. NA indicated that the exact case number was too small to be calculated

Furthermore, subgroup analyses were performed to find the best population benefit for Escalated‐R‐CHOP‐21. We implemented stratified logistic analysis in the matched elderly male cohort and the matched younger male cohort. Among patients older than 60 years of age, Escalated‐R‐CHOP‐21 treatment conferred a lower risk of disease progression and mortality, but younger men tended to benefit more from the Standard‐R regimen. Among the younger men, no significant difference between the Escalated‐R regimen and the Standard‐R regimen was observed in different subgroups (Figures 3 and 4, Figures S2 and S3).

Dose effects on PFS and OS with the Escalated‐R‐CHOP‐21 were observed in elderly men who had elevated pretreatment serum LDH (*p* = 0.016 and *p* = 0.017), no B symptom (*p* = 0.018 and *p* = 0.011), no high‐risk extranodal sites (*p* = 0.005 and *p* = 0.009), and no CNS prophylaxis (*p* = 0.014 and *p* = 0.018). Patients with higher stage, IPI, NCCN‐IPI, and CNS‐IPI index showed significant benefit from Escalated‐R‐CHOP‐21 in OS (*p* = 0.044, 0.043, 0.038, and 0.029). There was no statistical significance in the benefit of escalated‐dose rituximab compared to standard‐dose rituximab in either the GCB subtype or the non‐GCB subtype. There was a significant interaction between the high‐risk extranodal sites and treatment regimen for the PFS (interaction *p* = 0.030). Other than that, there were no significant interactions with each subgroup (interaction *p* > 0.05). Therefore, the dose effect in PFS of Escalated‐R‐CHOP‐21 was more obvious for elderly men with no high‐risk extranodal sites.

### 3.6 Toxicity

We also investigated the toxicity of Escalated‐R‐CHOP‐21 compared to Standard‐R‐CHOP‐21 in elderly men. The most common any‐grade adverse events were leukocytopenia (77.1%) and anemia (58.3%) in the Escalated‐R group, and the most common grade 3 and 4 adverse event was leukocytopenia (52.1%). There were no significant differences concerning any grade or grade 3 and 4 toxicities between the Escalated‐R group and the matched Standard‐R group. We found that 2.5% (5/201) of the elderly men had treatment‐related deaths, 2.1% (1/48) of patients in the Escalated‐R group, vs. 6.2% (3/48) of patients in the matched Standard‐R group (*p* = 0.617). More importantly, a tendency of increased interstitial pneumonia of any grade was observed in the Escalated‐R group, although the difference was not statistically significant (29.2% vs. 18.8%, *p* = 0.232) and grade 3 and 4 interstitial pneumonia did not increase in the Escalated‐R group (Table 4).

**TABLE 4:** Any grade or grade 3–4 toxicity during R‐CHOP‐21 regimen in the elderly male cohort

	Unmatched standard‐R group (*n* = 153)		Matched standard‐R group (*n* = 48)		Escalated‐R group (*n* = 48)	
	Any grade	Grade 3–4	Any grade	Grade 3–4	Any grade	Grade 3–4
Leukocytopenia^a^	127 (83.0%)	92 (60.1%)	40 (83.3%)	23 (47.9%)	37 (77.1%)	25 (52.1%)
Anemia^a^	101 (66.0%)	8 (5.2%)	34 (70.8%)	2 (4.2%)	28 (58.3%)	5 (10.4%)
Thrombocytopenia^a^	42 (27.5%)	13 (8.5%)	11 (22.9%)	3 (6.3%)	14 (29.2%)	6 (12.5%)
Pneumonia	60 (39.2%)	18 (11.8%)	20 (41.7%)	6 (12.5%)	17 (35.4%)	6 (12.5%)
Interstitial pneumonia	23 (15.0%)	8 (5.2%)	9 (18.8%)	4 (8.3%)	14 (29.2%)	6 (12.5%)
Arrhythmia	10 (6.5%)	3 (2.0%)	4 (8.3%)	1 (2.1%)	4 (8.3%)	0 (0%)
Neuropathy	11 (7.2%)	1 (0.7%)	3 (6.3%)	0 (0%)	3 (6.3%)	1 (2.1%)

Note

Data are numbers of patients had toxicity, with percentages in parentheses.

^a^ Represents hematological toxicities.

## 4 DISCUSSION

Rituximab has been successfully applied to treatment of non‐Hodgkin lymphoma for more than 20 years, and R‐CHOP has significantly increased the survival rate of DLBCL.^21^ Improving the efficacy by optimizing R‐CHOP as first‐line therapy for DLBCL is one of the priorities of the current research.^22^ Some studies have found that significant differences in the efficacy of RCHOP by age and sex, and elderly and male patients treated with R‐CHOP have worse survival than younger and female patients.^23^, ^24^ The faster clearance rate of rituximab in men might be a key determinant of the clinical efficacy of RCHOP treatment.^12^ Previous studies have demonstrated that increasing the rituximab dose resulted in higher serum levels and longer exposure times, and this translated into clinical outcome benefit in elderly men.^25^, ^26^ In this study, we aimed to investigate the possible benefit of increasing the rituximab dose with CHOP‐21 as the first‐line treatment in male patients with DLBCL. Our results showed that the PFS and OS of elderly men were worse than that of younger men for using Standard‐R‐CHOP‐21 (3‐year PFS: 61.7% vs. 78.0%, *p* < 0.001; 3‐year OS: 71.1% vs. 89.0%, *p* < 0.001). When using Escalated‐R‐CHOP‐21, the PFS and OS were slightly better for elderly men than for younger men (3‐year PFS: 75.5% vs. 65.6%, *p* = 0.177; 3‐year OS: 86.6% vs. 80.4%, *p* = 0.566). Because the results might be influenced by the differences in clinicopathological characteristics and prognostic factors between younger and older patients, we divided the patients into the younger and older group and used propensity score matching. We further conclude that the Escalated‐R‐CHOP‐21 probably improved treatment outcomes in elderly men with DLBCL, but not in younger men.

Based on the literature, the majority of the disease relapses occur within the 2 years after R‐CHOP as first‐line immunochemotherapy.^27^, ^28^ Therefore, it is appropriate to use 3‐year PFS and 3‐year OS to describe the survival benefit of Escalated‐R‐CHOP‐21. We found that the Escalated‐R‐CHOP‐21 schedule resulted in a significantly better outcome for elderly men than Standard‐R‐CHOP‐21 for 3‐year PFS (75.5% vs. 61.7%) and 3‐year OS (86.6% vs. 71.1%). The outcome in this study is similar to that of the SEIXE‐R‐CHOP‐14 trial.^9^ The SEIXE‐R‐CHOP‐14 used a planned historical comparison of patients from RICOVER‐60.^29^ It was concluded that 500 mg/m^2^ rituximab in SEIXE‐R‐CHOP‐14 was not more toxic than 375 mg/m^2^ rituximab in RICOVER‐60. Elderly men from SEIXE‐R‐CHOP‐14 received 500 mg/m^2^ improved the PFS (3‐year PFS: 76% vs. 68%) and had a trend for a better OS (3‐year OS: 79% and 73%) than elderly men from RICOVER‐60 375 mg/m^2^ rituximab.^9^

Nevertheless, the effect of rituximab dose escalation on R‐CHOP has remained controversial. The outcome was not significantly better when the entire study population in DENSE‐R was compared to RICOVER‐60.^25^ Even though patients with IPI 3–5 in DENSE‐R had better CR rates, it did not translate into better long‐term outcomes, just with a decreased male hazard. More particularly, elderly men with a poor prognosis (IPI of 3–5) benefited considerably more from the SMARTE‐R schedule.^10^ Notably, directly comparing these results to RICOVER‐60 without careful matching is slightly inappropriate, and several confounding factors could have influenced the results.

Given the current lack of high‐quality studies, the latest randomized phase III trial of HOVON‐84 was carried out to focus on increased rituximab frequency in patients with untreated DLBCL during R‐CHOP‐14.^30^ Patients in this study were randomly allocated to receive either R‐CHOP‐14 or R‐CHOP‐14 with an intensification of rituximab in the first four cycles (RR‐CHOP‐14). The impact of RR‐CHOP‐14 vs. R‐CHOP‐14 on FFS, PFS, DFS, and OS was not different between subgroups of age (18–65 vs. 66–80 years), sex (male vs. female), or age‐adjusted IPI score. This study found that early rituximab intensification does not improve the outcome of DLBCL. Although the results of the randomized phase III trial are more convincing, they still did not focus on elderly men. The subgroup analyses of old age included elderly women and the analyses of sex included younger men who might not benefit from the intensification of rituximab. However, elderly men, the most meaningful group, were not considered.

In our study, we found that younger men could not obtain a better outcome with increased rituximab doses. On the contrary, the Standard‐R‐CHOP‐21 schedule resulted in a slightly better outcome than the Escalated‐R‐CHOP‐21 for 3‐year PFS (82.2% vs. 65.6%, *p* = 0.087) and 3‐year OS (87.6% vs. 80.4%, *p* = 0.397) in the matched younger male cohort. The reasons for these results were ultimately unclear. The baseline characteristics of patients in both groups were well balanced before and after matching. We think the most possible reason was the different rituximab pharmacokinetics. The median rituximab clearance in younger men (9.89 ml/h) was lower in than elderly men (10.59 ml/h).^12^ The Escalated‐R dose may not be translated into survival benefits rather seemed to increase the toxicity in younger men. The HOVON‐84 study had shown that the PFS of RCHOP tended to be superior to the PFS of RR‐CHOP, this phenomenon is more obvious in younger patients.^30^ The HOVON‐84 study did not explain the reasons except mentioning that the rituximab levels cannot be converted into survival. In addition, there are other possible reasons including the different genotypes, the age‐specific role of the immune system, the association between the encompassing drug absorption, drug distribution, and drug metabolism. Patient selection bias may be existed. All in all, compared to the elderly men, younger men had probably not benefited from the high dose of rituximab.

We also paid more attention to identifying and finding subpopulations who benefited from an increasing rituximab dose as first‐line treatment in our study. For the first time, we found that elderly men with an elevated LDH level could benefit from Escalated‐R‐CHOP‐21. Elevated LDH is associated with aggressiveness, resistance to chemotherapy, and poor survival of DLBCL.^31^, ^32^ Moreover, elevated LDH is also associated with a high total metabolic tumor volume (TMTV), which is related to worse PFS and OS in DLBCL.^33^, ^34^ Rituximab exposure is influenced by TMTV and correlates with the response and outcome of DLBCL.^35^ We believe that a higher LDH level might indicate a lower rituximab exposure, and this can be rescued by increasing the rituximab dose. Notably, we found that patients with no high‐risk extranodal sites had significantly benefited from Escalated‐R‐CHOP‐21 in PFS and OS. A study conducted by Dr. Castillo found that extranodal primary sites were more likely to present in patients with early‐stage.^36^ In contrast, our study found that high‐risk extranodal sites were associated with higher stage and higher LDH. The possible reason for this discrepancy is difficult to obtain, but may be due to the small sample size or the difference of rituximab exposure. We could not directly confirm this hypothesis because no assessment of the pharmacokinetics was undertaken, so further investigation will be necessary to confirm this conjecture.

Even though LDH accounted for part of IPI, higher IPI (3–5) and lower IPI (0–2) did not observe differences of PFS between Escalated‐R‐CHOP‐21 and Standard‐R‐CHOP‐21 in this study. However, in the elderly men of higher IPI (3–5), there was a significant improvement in OS for Escalated‐R‐CHOP‐21. These results were consistent with the previous study.^10^ The SMARTE‐R schedule resulted in a significantly better outcome for IPI of 3–5 compared with that in RICOVER‐60 in subgroup analysis. Fifty‐one elderly men with poor prognosis (IPI 3–5) benefited considerably more from the SMARTE‐R than 66 elderly men with IPI 3–5 in RICOVER‐60 in 3‐year EFS (67% vs. 43%), 3‐year PFS (71% vs. 53%), and 3‐year OS (80% vs. 60%).^10^ We identified that the patients with lower IPI were more highly chosen for using Escalated‐R‐CHOP‐21 in elderly men. Differently, almost all elderly patients were generally identified with higher IPI in the published literature.^22^, ^37^ This probably underlies the discrepancy of the patients’ selection.

The incidence of CNS relapse was very low (12/522, 2.3%) in our study. The low‐risk group, the intermediate‐risk group, and the high‐risk group for CNS recurrence identified by CNS‐IPI showed that 3‐year rates of CNS disease were 1.2%, 1.4%, and 10.1% in our study, which was similar with the previous study.^16^ Patients with high‐risk factors generally received more prophylaxis (low‐risk: 6.7%; intermediate‐risk: 10.5%; and high‐risk: 31.9%), but the 3‐year CNS recurrence rate of the high‐risk group still reached 10.1%. High‐dose rituximab was not significantly associated with CNS relapse in all patients (HR: 0.335, *p* = 0.296) or the low‐risk group (HR: 0.035, *p* = 0.559), the intermediate‐risk group (HR: 0.031, *p* = 0.606), and the high‐risk group (HR: 0.849, *p* = 0.882), respectively. CNS prophylaxis for DLBCL still remains controversial. Most studies observed no clear benefit for prophylaxis in the era of R‐CHOP according to CNS‐IPI.^38^, ^39^ The last study indicated that the benefit of HD‐MTX for CNS prophylaxis is transient.^18^ The incidence of CNS relapse was very low (21/1080, 1.9%) confirming the reduced incidence in the rituximab era, and the incidence was 2.8% for patients selected to receive prophylaxis, so there is very limited use of prophylaxis.^40^ Therefore, we are not particularly concerned with the influence of CNS prophylaxis due to the limitation of data.

This study has several limitations that should be mentioned. First, the sample size is small, and the subgroup analyses need to be validated in a larger group. Second, the results of this study cannot avoid the existence of selection bias. The relatively better outcomes for the patients might be affected by their general condition. It seems that elderly patients with better conditions (lower IPI and less high‐risk extranodal sites) were more likely to receive high‐dose treatment in this study. However, the condition of patients was adjusted in the propensity score matching and Cox‐regression analysis, which partially eliminated the selection bias. Third, only individuals from our center were included; whether these findings can be transferable to other hospitals and regions were not determined. Fourth, toxicity analysis was limited and difficult to obtain because it is retrospectively collected from medical records. So further investigations of multicenter prospective study are needed and which may be helpful to identify the potential therapeutic population treated with Escalated‐R‐CHOP‐21.

In conclusion, our study showed that the Escalated‐R‐CHOP‐21 may be an effective and safe front‐line regimen with the improvement of long‐term survival in elderly men with DLBCL. This study provides new insight into the clinical application of the high‐dose rituximab combined with CHOP‐21 in DLBCL.

## ETHICS STATEMENT

The studies involving human participants were reviewed and approved by the Institutional Ethical Boards of Sun Yat‐sen University Cancer Center (No: B2020‐279‐01).

## CONFLICT OF INTEREST

The authors have declared no conflict of interest.

## Supporting information

Supplementary Material

## Data Availability

All datasets used and/or analyzed during this study are available in the Research Data Deposit public platform (www.researchdata.org.cn, number RDDA2021156058) from the corresponding author upon reasonable request.
