# Supplementary material for: The beneficial effect of Escalated‐R‐CHOP‐21 for the treatment of diffuse large B‐cell lymphoma in elderly male patients: A propensity‐matched cohort study
Source: Cancer Med. 2021 Sep 28;10(21):7650–64. doi: 10.1002/cam4.4296 (PMC8559475; doi:10.1002/cam4.4296)
Supplement: Supplementary file 1 — Supplementary Material [file CAM4-10-7650-s001.pdf]

Supplementary Material

1 Supplementary Figures and Tables

1.1 Supplementary Figures

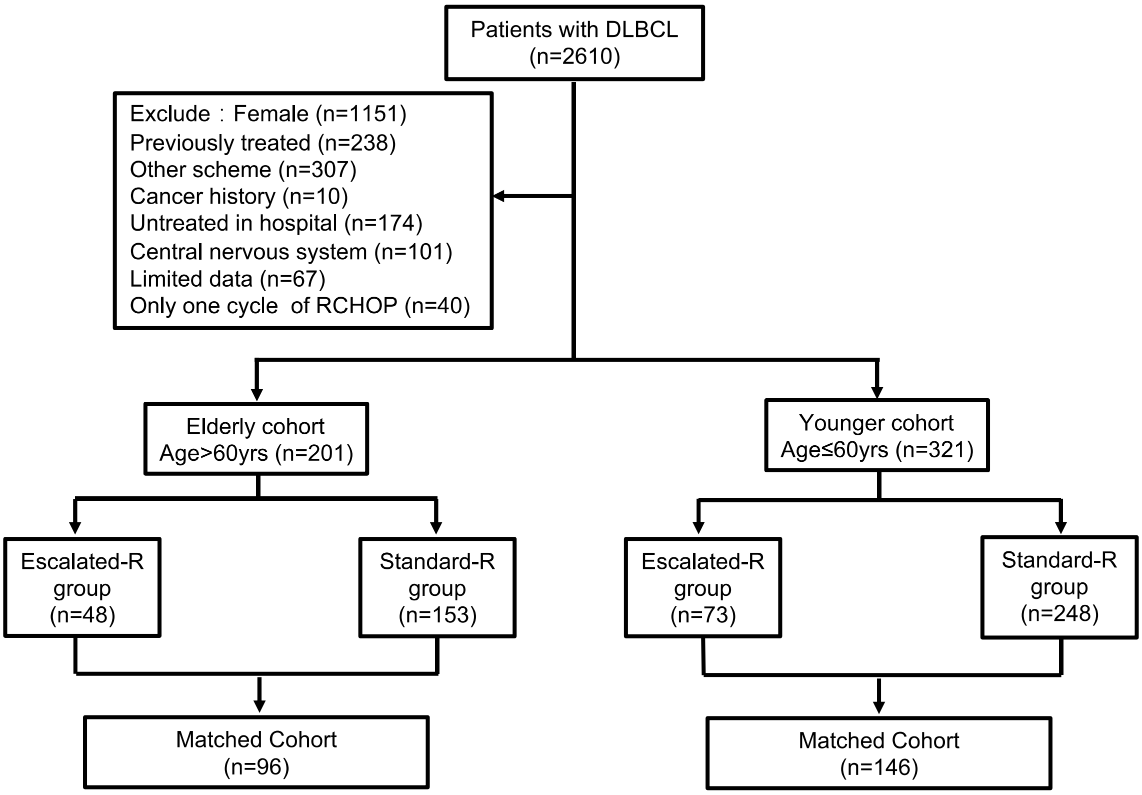

Supplementary Figure 1 Patient's flow chart. Escalated-R group means that the patients who used Escalated-R-CHOP-21, and Standard-R group means that the patients who used Standard-R-CHOP-21

Total patients	Hazard Ratio (95%CI)	Effect P value	Interaction P value
**age>60 (n=96)**			
B symptom			0.346
No	0.37 (0.16-0.84)	**0.018**	
Yes	0.82 (0.18-3.65)	0.790	
Cell of origin			0.567
GCB	0.31 (0.08-1.13)	0.076	
Non-GCB	0.59 (0.24-1.44)	0.248	
CNS prophylaxis			0.257
No	0.34 (0.14-0.80)	**0.014**	
Yes	0.89 (0.20-3.99)	0.880	
CNS-IPI			0.757
Low (0-1)	0.31 (0.06-1.54)	0.153	
High ≥2	0.46 (0.20-1.04)	0.062	
**age≤60 (n=146)**			
B symptom			0.306
No	1.47 (0.68-3.15)	0.327	
Yes	2.43 (0.75-7.92)	0.140	
Cell of origin			0.522
GCB	1.15 (0.42-3.19)	0.784	
Non-GCB	2.03 (0.90-4.60)	0.089	
CNS prophylaxis			0.591
No	2.07 (0.99-4.30)	0.052	
Yes	1.12 (0.27-4.68)	0.882	
CNS-IPI			0.980
Low (0-1)	2.20 (0.79-6.13)	0.132	
High ≥2	1.53 (0.68-3.45)	0.306	

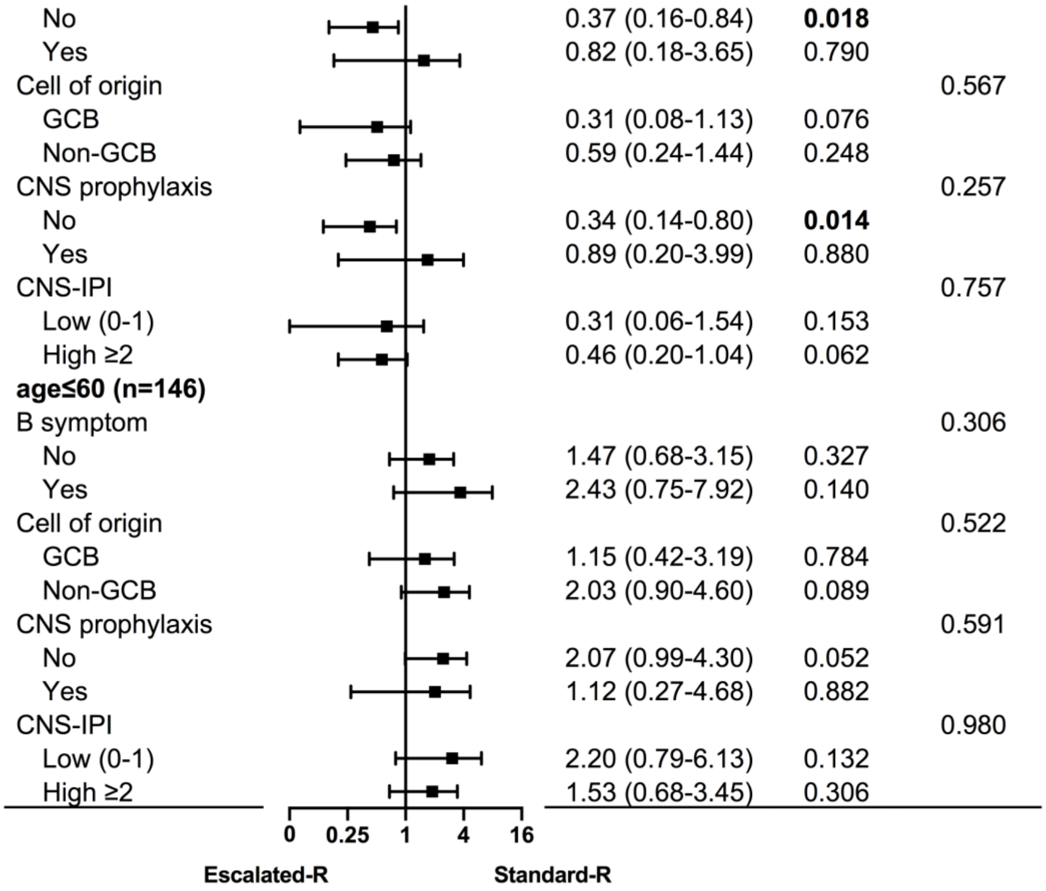

Supplementary Figure 2 Forest plot for another subgroup analyses of PFS according to the age of male patients. The Escalated-R group was compared with the Standard-R group in calculating hazard ratios and 95% confidence intervals.

Total patients	Hazard Ratio (95%CI)	Effect P value	Interaction P value
**age>60 (n=96)**			
B symptom			0.194
No	0.27 (0.10-0.74)	**0.011**	
Yes	1.28 (0.18-9.13)	0.803	
Cell of origin			0.779
GCB	0.28 (0.06-1.33)	0.108	
Non-GCB	0.46 (0.16-1.36)	0.162	
CNS prophylaxis			0.324
No	0.30 (0.11-0.81)	**0.018**	
Yes	0.79 (0.11-5.64)	0.814	
CNS-IPI			0.700
Low (0-1)	0.49 (0.09-2.64)	0.409	
High ≥2	0.32 (0.11-0.89)	**0.029**	
**age≤60 (n=146)**			
B symptom			0.143
No	0.92 (0.31-2.67)	0.872	
Yes	2.71 (0.70-10.48)	0.149	
Cell of origin			0.477
GCB	0.85 (0.22-3.21)	0.808	
Non-GCB	1.86 (0.66-5.23)	0.242	
CNS prophylaxis			0.255
No	1.99 (0.78-5.08)	0.151	
Yes	0.43 (0.05-3.87)	0.453	
CNS-IPI			0.063
Low (0-1)	0.55 (0.10-2.92)	0.482	
High ≥2	2.23 (0.82-6.03)	0.116	

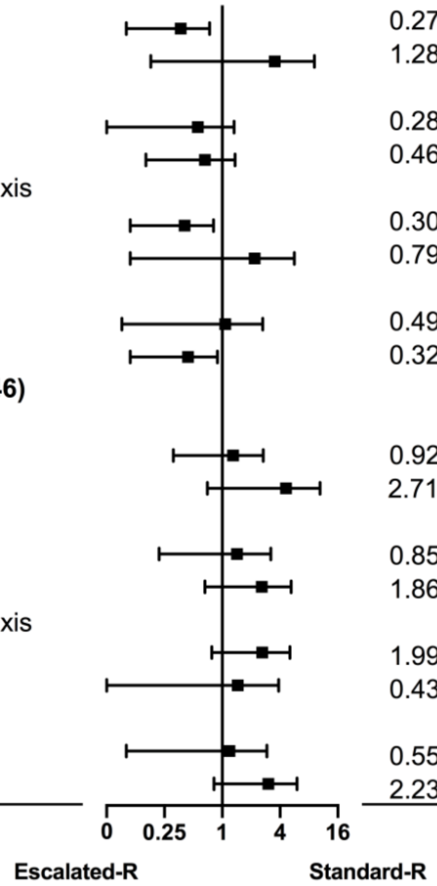

Supplementary Figure 3 Forest plot for another subgroup analyses of OS according to the age of male patients. The Escalated-R group was compared with the Standard-R group in calculating hazard ratios and 95% confidence intervals.

1.2 Supplementary Tables

Supplementary Table 1. Baseline participant characteristics of the younger male cohort.

	Unmatched younger Cohort			Matched younger Cohort		
	Standard-R(n=248)	Escalated-R(n=73)	P value	Standard-R(n=73)	Escalated-R(n=73)	P value
Age(y)	43.4 ± 11.4	44.8 ± 11.1	0.326	43.4 ± 11.2	44.8 ± 11.1	0.450
Stage			0.425			1
I-II	148 (59.7)	40 (54.8)		40 (54.8)	40 (54.8)	
III-IV	100 (40.3)	33 (45.2)		33 (45.2)	33 (45.2)	
Extranodal sites			0.354			1
≤1	193 (77.8)	53 (72.6)		53 (72.6)	53 (72.6)	
>1	55 (22.2)	20 (27.4)		20 (27.4)	20 (27.4)	
PS			0.702			1
0-1	234 (94.4)	68 (93.2)		68 (93.2)	68 (93.2)	
2-4	14 (5.6)	5 (6.8)		5 (6.8)	5 (6.8)	
LDH(U/L)			**0.034**			1
≤250	163 (65.7)	38 (52.1)		38 (52.1)	38 (52.1)	
>250	85 (34.3)	35 (47.9)		35 (47.9)	35 (47.9)	
B symptom			0.288			0.689
No	204 (82.3)	56 (76.7)		58 (79.5)	56 (76.7)	
Yes	44 (17.7)	17 (23.3)		15 (20.5)	17 (23.3)	
Bulky disease			0.366			0.093
≤5cm	169 (68.1)	52 (71.2)		43 (58.9)	52 (71.2)	
5cm<x≤10cm	54 (21.8)	11 (15.1)		22 (30.1)	11 (15.1)	
>10cm	25 (10.1)	10 (13.7)		8 (11.0)	10 (13.7)	
Cell of origin			0.421			0.393
GCB	101 (40.7)	34 (46.6)		33 (45.2)	34 (46.6)	
Non-GCB	137 (55.2)	38 (52.1)		36 (49.3)	38 (52.1)	
Unclassified	10 (4.0)	1 (1.4)		4 (5.5)	1 (1.4)	
Primary sites			0.706			0.771
Nodal	136 (54.8)	36 (49.3)		39 (53.4)	36 (49.3)	
Extranodal, high-risk	72 (29.0)	24 (32.9)		20 (27.4)	24 (32.9)	
Extranodal, other	40 (16.1)	13 (17.8)		14 (19.2)	13 (17.8)	
CNS prophylaxis			0.751			0.099
No	221 (89.1)	66 (90.4)		59 (80.8)	66 (90.4)	
Yes	27 (10.9)	7 (9.6)		14 (19.2)	7 (9.6)	
CNS-IPI			0.345			0.458
Low	173 (69.8)	47 (64.4)		47 (64.4)	47 (64.4)	
Intermediate	52 (21.0)	21 (28.8)		17 (23.3)	21 (28.8)	
High	23 (9.3)	5 (6.8)		9 (12.3)	5 (6.8)	
IPI			0.495			1
Low	173 (69.8)	47 (64.3)		47 (64.4)	47 (64.4)	
LI	39 (15.7)	10 (13.7)		10 (13.7)	10 (13.7)	
HI	28 (11.3)	13 (17.8)		13 (17.8)	13 (17.8)	

High	8 (3.2)	3 (4.1)		3 (4.1)	3 (4.1)	
NCCN-IPI			0.284			0.880
Low	112 (45.2)	32 (43.8)		30 (41.1)	32 (43.8)	
LI	113 (45.5)	29 (39.7)		32 (43.8)	29 (39.7)	
HI	22 (8.9)	12 (16.4)		11 (15.1)	12 (16.4)	
High	1 (0.4)	0		0	0	

Note: Data of age are means ± standard deviations and p-value computed using an independent sample t-test for continuous variables. Numbers of patients with percentages in parentheses and p-value computed using the chi-square test or fisher exact test for categorical variables. PS, performance state. LDH, lactic dehydrogenase. IPI: Low risk:0-1, Intermediate low (LI):2, Intermediate high (HI):3, High risk: ≥4. NCCNIPI: Low risk:0-1, Intermediate low (LI):2-3, Intermediate high (HI):4-5, High risk: ≥6.

Supplementary Table 2. Univariable and Multivariable analyses of prognostic factors for PFS and OS in the younger male cohort.

	PFS		OS	
	HR (95%CI)	P value	HR (95%CI)	P value
**Univariate analysis**				
Stage	2.86		2.35	
III-IV vs. I-II	1.84-4.43	**<0.001**	1.30-4.25	**0.005**
Extranodal sites	3.40		2.73	
>1 vs. ≤1	2.21-5.23	**<0.001**	1.51-4.91	**0.001**
PS	5.08		5.85	
2-4 vs. 0-1	2.86-9.04	**<0.001**	2.89-11.81	**<0.001**
LDH (U/L)	3.49		5.06	
>250 vs. ≤250	2.26-5.40	**<0.001**	2.66-9.62	**<0.001**
Bulky disease	1.35		1.57	
>10 vs. 5-10 vs. ≤5	1.02-1.79	**0.035**	1.09-2.27	**0.015**
B symptom	1.89		2.47	
Yes vs. No	1.18-3.03	**0.008**	1.35-4.53	**0.004**
Cell of origin	1.89		2.04	
Non-GCB vs. GCB	1.20-3.00	**0.007**	1.08-3.88	**0.029**
Primary sites	1.51	0.069	1.85	**0.041**
Extranodal, high-risk vs. Others	0.97-2.34		1.03-3.33	
CNS prophylaxis	2.26	**0.003**	1.88	0.106
Yes vs. No	1.31-3.90		0.88-4.02	
CNS-IPI	3.66	**<0.001**	3.28	**<0.001**
High vs. Low	2.39-5.62		1.83-5.88	
IPI	4.31		4.57	
High vs. Low	2.75-6.74	**<0.001**	2.54-8.24	**<0.001**
NCCN-IPI	3.03		3.35	
High vs. Low	1.78-5.17	**<0.001**	1.70-6.61	**<0.001**

R dosage	1.53		1.63	
Escalated vs. Standard	0.95-2.45	0.081	0.85-3.12	0.138
**Multivariate analysis with exposure†**				
R dosage	1.39		1.33	
Escalated vs. Standard	0.86-2.25	0.180	0.68-2.60	0.398
**Multivariate analysis with IPI**				
R dosage	1.40		1.52	
Escalated vs. Standard	0.87-2.26	0.165	0.79-2.92	0.207
**Multivariate analysis with NCCN-IPI**				
R dosage	1.47		1.54	
Escalated vs. Standard	0.91-2.36	0.115	0.80-2.95	0.193
**Multivariate analysis with CNS-IPI**				
R dosage	1.46		1.61	
Escalated vs. Standard	0.91-2.35	0.115	0.84-3.07	0.151

Note: P-value <0.05 in bold are statistically significant. †Represents variables with P value <0.1 in the univariate analysis except for CNS-IPI, IPI and NCCNIPI. PS, performance state. LDH, lactic dehydrogenase. High and low CNS-IPI was defined as the risk score 0-1 and more than 2, respectively. High and low IPI was defined as the risk score 0-2 and 3-5, respectively. High and low NCCNIPI was defined as the risk score 0-3 and more than 4, respectively.
